# Effect of reduced energy density of close-up diets on metabolites, lipolysis and gluconeogenesis in Holstein cows

**DOI:** 10.5713/ajas.18.0624

**Published:** 2019-01-04

**Authors:** Wenming Huang, Libin Wang, Shengli Li, Zhijun Cao

**Affiliations:** 1 Department of Animal Science, College of Animal Science, Southwest University, Chongqing 402460, China; 2 State Key Laboratory of Animal Nutrition, College of Animal Science and Technology, China Agricultural University, Beijing 100193, China

**Keywords:** Transition Cow, Dietary Energy Density, Lipolysis, Gluconeogenesis, Blood Metabolites

## Abstract

**Objective:**

An experiment was conducted to determine the effect of reduced energy density of close-up diets on metabolites, lipolysis and gluconeogenesis in cows during the transition period.

**Methods:**

Thirty-nine Holstein dry cows were blocked and assigned randomly to three groups, fed a high energy density diet (HD, 1.62 Mcal of net energy for lactation [NE_L_]/kg dry matter [DM]), a medium energy density diet (MD, 1.47 Mcal NE_L_/kg DM), or a low energy density diet (LD, 1.30 Mcal NE_L_/kg DM) prepartum; they were fed the same lactation diet to 28 days in milk (DIM). All the cows were housed in a free-stall barn and fed *ad libitum*.

**Results:**

The reduced energy density diets decreased the blood insulin concentration and increased nonesterified fatty acids (NEFA) concentration in the prepartum period (p<0.05). They also increased the concentrations of glucose, insulin and glucagon, and decreased the concentrations of NEFA and β-hydroxybutyrate during the first 2 weeks of lactation (p<0.05). The plasma urea nitrogen concentration of both prepartum and postpartum was not affected by dietary energy density (p>0.05). The dietary energy density had no effect on mRNA abundance of insulin receptors, leptin and peroxisome proliferator-activated receptor-γ in adipose tissue, and phosphoenolpyruvate carboxykinase, carnitine palmitoyltransferase-1 and peroxisome proliferator-activated receptor-α in liver during the transition period (p>0.05). The HD cows had higher mRNA abundance of hormone-sensitive lipase at 3 DIM compared with the MD cows and LD cows (p = 0.001). The mRNA abundance of hepatic pyruvate carboxykinase at 3 DIM tended to be increased by the reduced energy density of the close-up diets (p = 0.08).

**Conclusion:**

The reduced energy density diet prepartum was effective in controlling adipose tissue mobilization and improving the capacity of hepatic gluconeogenesis postpartum.

## INTRODUCTION

The transition period, from 21 d before to 21 d after parturition, is the most stressful time in the production cycle of dairy cows because of depressed dry matter intake (DMI) and marked changes in metabolism that support late gestation, parturition and the onset of milk synthesis [1–3]. The abrupt increase in energy demand and low DMI after parturition result in a negative energy balance (NEB). The DMI rather than milk yield is the major driver of NEB [4]. Therefore, nutritional management during the transition period designed to increase postpartum DMI may be a potential strategy to alleviate the negative effect of NEB on performance, as well as related metabolic disorders. Efficient gluconeogenesis is the major pathway used by dairy cows to maintain an adequate glucose supply to the mammary gland [5,6]. When gluconeogenesis is insufficient, mobilization of body fat reserves is the main way used to meet the energy requirement of high milk production during early lactation. Blood nonesterified fatty acids (NEFA) concentrations are used as an indirect measure of the mobilization of triacylglycerol from adipose tissue [7], and NEFA are taken up proportionally by the liver [8]. Extensive lipolysis is associated with postpartum metabolic disorders such as fatty liver and ketosis [9,10]. Fatty liver reduces the capacity for liver gluconeogenesis, and this will intensify NEB and lipolysis.

Earlier strategies focused on feeding energy-dense diets and maximizing DMI prepartum; they were aimed at supporting development of the conceptus and mammary gland, and preparing dry cows for better adaptation to the early-lactation diet [7,11]. In contrast to this viewpoint, in the past 15 years, some studies have shown that overconsumption of energy prepartum often results in a slower increase in DMI postpartum when compared with cows on a restricted intake [12–14] or those fed on a low energy density diet containing wheat straw [10]. Most of the above studies used moderate or high energy density diets with restricted DMI to control energy intake, or the cows were housed in a tie-stall barn. However, cows in the transition period on commercial farms normally have bunk space with free access to feed and water, instead of being restricted in a tie-stall barn with limited access to feed.

It is very common to use moderate or even high energy density transition diets on Chinese dairy farms and the incidence of metabolic diseases is sometimes very high. However, China is short of high quality forage and abundant in low quality forage (*Leymus chinensis* hay, corn stover and wheat straw). In our previous study, the prepartum dairy cows fed lower energy diets containing more *Leymus chinensis* hay had lower DMI and energy intake prepartum, but had higher milk yield and DMI postpartum, and lost numerically less body weight (BW) and body condition score (BCS) postpartum, compared with dairy cows fed higher energy diets prepartum [3]. The objective of this study was to investigate further the effect of dietary energy density in the close-up dry period (the last 3 wks prepartum) on blood metabolites related to energy balance (EB), and the mRNA abundance of rate-limiting enzymes with regard to lipolysis and gluconeogenesis for multiparous Holstein cows fed *ad libitum* and housed in a free-stall barn.

## MATERIALS AND METHODS

### Animal care

Animal care and use were approved and conducted in accordance with the practices outlined in the Guide for the Care and Use of Agriculture Animals in Agriculture Research and Teaching [15].

### Animals and design

Thirty-nine multiparous Holstein cows were grouped according to milk production in the first 3 months of the previous parity, parity, BW, BCS and expected calving date, and assigned randomly to one of three dietary treatments. From dry-off to 22 d before expected parturition, all cows were fed the same far-off diet (net energy for lactation [NE_L_] 1.30 Mcal/kg dry matter [DM], Table 1). From 21 d before the expected day of calving until parturition, the cows were blocked and assigned randomly to three groups, fed a high energy density diet (HD treatment, n = 13; 1.62 Mcal NE_L_/kg DM; 14.0% crude protein [CP]), a medium energy density diet (MD treatment, n = 13; 1.47 Mcal NE_L_/kg DM; 14.0% CP), or a low energy density diet (LD treatment, n = 13; 1.30 Mcal NE_L_/kg DM; 14.0% CP; Table 1). Different amounts of water were added to each of the three diets to adjust the DM content to within 51% to 52%. After parturition, all the cows were provided with the same lactation diet (Table 1) to 28 day in milk (DIM).

The details of animal management and diets were the same as in our previous research [3]. In brief, cows were housed in a free-stall barn with a delivery room, and were fed the diets as a total mixed ration *ad libitum* throughout the experiment. Feed was offered once daily (at 16.00 hours) prepartum, and twice (at 07.30 and 14.30 hours) postpartum. Cows were milked three times daily.

### Blood collection and analyses of metabolites

Blood samples were obtained from the coccygeal vessel at 0900 h on d −21, −14, −7, 7, 14, 21, and 28 relative to parturition, and immediately after parturition. Blood was taken into evacuated serum separator tubes containing clot activator and into evacuated plasma tubes containing ethylenediaminetetraacetic acid. Tubes for plasma preparation were put on ice immediately after sampling and centrifuged at 3,000×*g* for 15 min at 4°C. Tubes for serum preparation were allowed to clot for 30 min at room temperature before centrifugation for 15 min at 2,000×*g* at 4°C. Plasma and serum were aliquoted into 1.5-mL centrifuge tubes and stored at −20°C until analysis. Plasma samples were sent to a clinical laboratory of the 309th Hospital (Beijing, China) for analysis of glucose, NEFA, and plasma urea nitrogen (PUN) using an autoanalyzer (ILAB 600, Instrumentation Laboratory, Lexington, MA, USA) with kits supplied by Instrumentation Laboratory. Plasma concentrations of β-hydroxybutyrate (BHBA) were measured using a BHBA kit (Sigma-Aldrich Chemical Co., Shanghai, China). Radioimmunoassay kits were used to measure serum insulin (Coat-A-Count Insulin, Siemens Medical Solutions USA Inc., Malvern, PA, USA) and plasma glucagon (Double Antibody Glucagon, Siemens Medical Solutions USA Inc., USA).

### Liver and adipose tissue collection and quantitative polymerase chain reaction analysis

Five cows per treatment were selected randomly for collection of liver and adipose tissue at 1000 h on d −7, 3, and 14 relative to parturition. Liver tissue was sampled via puncture biopsy under general anesthesia and local anesthesia as described by Zom et al [16]. Subsequently, adipose tissue was sampled from the tail region between the ischium pin bone and coccygeal vertebrae as described by Goselink et al [17]. All dissected liver and adipose tissue samples were washed with normal saline and immediately snap frozen in liquid nitrogen and stored at −80°C until RNA extraction.

For measurement of gene expression, frozen samples of liver and adipose tissue were ground under liquid nitrogen and total RNA was isolated using TRIzol reagent (Invitrogen, Breda, the Netherlands), following the manufacturer’s instructions. To eliminate DNA contamination, the isolated RNA was subjected to an on-column DNase treatment (NucleoSpin RNA II kit; Macherey-Nagel GmbH & Co. KG, Duren, Germany). Reverse transcription of 1 μg of total RNA was performed in a 20-μL reaction using Superscript III reverse transcriptase (Invitrogen, the Netherlands) according to the manufacturer’s protocol. The mRNA expression was assessed using a SYBR Premix Ex Taq II kit (TaKaRa Biotechnology Co. Ltd., Dalian, China), following the instructions. The reactions were run in triplicate on a 7500 Fast Real-Time PCR System (Applied Biosystems Deutschland GmbH, Darmstadt, Germany), and the relative expression of genes was analyzed using the 2^−ΔΔCT^ method [18].

We measured the transcript levels of the following key enzymes related to fatty acid and energy metabolism in the liver: carnitine palmitoyltransferase-1 (CPT1), peroxisome proliferator-activated receptor-α (PPARα), pyruvate carboxykinase (PC), and phosphoenolpyruvate carboxykinase (PEPCK). In addition, the expression of hormone sensitive lipase (HSL), peroxisome proliferator-activated receptor-γ (PPARγ), insulin receptor (InsR), and leptin was analyzed in adipose tissue. All the primers are presented in Table 2. Housekeeping genes ribosomal protein L19 (RPL19) and β-actin were analyzed as internal standards.

### Statistical analysis

To avoid problems with fitting the covariance structure, data for blood parameters were analyzed separately for the prepartum and postpartum periods. The data for blood parameters were evaluated using the MIXED procedure of SPSS 16.0 (SPSS Inc., Chicago, IL, USA) for repeated measures with the following model: Y_ikn_ = μ+W_i_+T_k_+WT_ik_+C_(ik)n_, where Y_ikn_ = an observation from the ith week relative to calving, kth treatment, and nth cow; μ = the grand mean; W_i_ = effect of the ith week; T_k_ = effect of the kth treatment; WT_ik_ = effect of the week by treatment interaction; and C_(ik)n_ = random experimental error from the nth cow nested within the ith week and kth treatment. A repeated statement was used for variables measured over time. Covariance structures including first-order autoregressive (AR[1]), compound symmetry, and unstructured were tested. AR(1) yielded the lowest Akaike’s criterion and was finally used in our model. The data on gene expression were evaluated by the one-way ANOVA procedure of SPSS 16.0 (SPSS Inc., USA). Tukey’s procedure for comparison of multiple means was used to separate treatment means. Least squares means were computed and are presented throughout. A value of p<0.05 was set as the significance level.

## RESULTS

### Blood metabolites and hormones

The blood glucose concentration both prepartum and on the day of calving was not affected by the dietary energy density (p>0.05; Table 3). The glucose concentration for the HD treatment was lower than for the MD treatment (p<0.05), and numerically lower than that for the LD treatment (p>0.05) during the first 4 wk of lactation. The prepartum concentration of insulin for the LD treatment was lower than that of the HD and MD treatments (p<0.05). After parturition, the LD and MD treatments gave higher insulin concentrations during the first 2 wk of lactation (p<0.05), but there was no significant difference among the three treatments during the first 4 wk of lactation (p>0.05). There was no significant difference among the three treatments in glucagon concentration before calving and on the day of calving (p>0.05). The glucagon concentration for the HD treatment was lower than those for the MD and LD treatments during the first 2 wk of lactation (p< 0.05), but there was no significant difference among the three treatments during the first 4 wk of lactation (p>0.05).

The LD treatment had higher prepartum NEFA concentration when compared with HD treatment (p<0.05). After parturition, the LD treatment had lower NEFA concentration when compared with the HD treatment during the first 2 wk and 4 wk of lactation (p<0.05). There was no significant difference among the three treatments in BHBA concentration before calving and on the day of calving (p>0.05). The HD treatment gave higher BHBA concentration than the MD treatment and LD treatment during the first 2 wk of lactation (p< 0.05), but there was no significant difference among the three treatments during the first 4 wk of lactation (p>0.05). The PUN concentration both prepartum and postpartum was not affect by dietary energy density (p>0.05).

### Expression of genes related to lipolysis and gluconeogenesis

The dietary energy density had no effect on the mRNA abundance of InsR, leptin, and PPARγ in adipose tissue both prepartum and postpartum (p>0.05; Table 4). The HD treatment gave higher mRNA abundance of HSL at 3 DIM compared with the MD and LD treatments (p = 0.001), but there was no significant difference at 7 d before calving and 14 DIM among the three treatments (p>0.05). The mRNA abundance of hepatic PEPCK, PC, CPT1, and PPARα, both prepartum and postpartum, was not affected by dietary energy density (p>0.05; Table 5). The mRNA abundance of hepatic PC at 3 DIM tended to be increased by the reduced energy density of close-up diets (p = 0.08).

## DISCUSSION

### Blood metabolites and hormones

#### Prepartum

Diets containing a greater amount of fermentable starch can result in increased blood glucose concentration in cattle. Some studies have demonstrated that prepartum dairy cows fed higher energy diets had higher blood glucose concentrations compared with those undergoing restriction of energy intake [10,14,19]. In the current study, the dietary energy density did not significantly affect prepartum blood glucose concentrations. Our previous study reported that cows in all three treatment groups offered *ad libitum* intake during the close-up period consumed more energy relative to their requirements [3]. The positive EB prepartum could explain why there was no significant difference in blood glucose concentrations among the three treatments. The prepartum concentration of insulin in LD cows was lower than that in HD and MD cows. Other studies have confirmed that cows had higher blood glucose and insulin concentrations in response to greater energy intake prepartum [10,19].

The prepartum concentrations of NEFA and postpartum NEFA and BHBA are usually considered important indicators of health condition and to predict disease occurrence during the periparturient period [20]. Circulating NEFA are used as an indirect measure of mobilization of triacylglycerol from adipose tissue. Compared with HD cows, the LD cows had higher blood NEFA concentration prepartum. The HD cows had higher energy intake and improved EB prepartum, which subsequently reduced fat mobilization. Others have also demonstrated that overfed cows had lower NEFA concentration than cows on restricted feed or those fed *ad libitum* a high forage diet [10,19]. The present study and others have noted that prepartum energy intake does not affect prepartum BHBA concentration, even in cows with restricted intake [10,14]. Douglas et al [21] reported that supplementation of fat, compared with a moderately high grain diet, did not affect prepartum blood BHBA concentration. The duration of feeding a high energy diet had no effect on blood BHBA concentration prepartum [7]. These results suggested that prepartum BHBA content for dairy cows was not affected by energy intake during the dry period.

#### Postpartum

All cows experienced a striking increase in blood glucose concentration at calving. Other authors have reported similar results [10,22]. Janovick et al [10] found that primiparous cows had lower blood glucose concentrations at parturition compared with multiparous cows, and this helped to explain the association of calving difficulty with blood glucose content. In our study, after parturition, HD cows had lower blood glucose concentrations than MD cows during the first 4 wk of lactation, and this was consistent with our previous study in which HD cows had lower lactose production and numerically lower DMI postpartum [3].

The blood insulin concentration decreased sharply at parturition for all groups in the present study, which coincided with an increased blood glucose concentration. This may be a mechanism to increase blood glucose concentration for calving. After parturition, although the insulin concentration for the three groups increased compared with the calving day, it was lower than the prepartum level. Alharthi et al [23] found both high and low BCS transition cows had lower plasma insulin concentrations on 7 and 20 DIM compared with 10 d before calving. Salin et al [24] suggested that the decrease in insulin concentration near calving is a major factor directing glucose to the mammary gland.

The NEB of early lactation is accompanied by an insulin-resistant state in tissues such as adipose tissue and skeletal muscle to ensure that nutrients are spared to provide an adequate supply to the mammary gland. To a certain extent, therefore, a degree of insulin resistance is needed to support higher milk production in early lactation [10]. Overfeeding energy prepartum has been demonstrated to decrease the glucose clearance rate in response to a glucose challenge administered at 3 wk postpartum, when compared with cows whose intake was controlled or restricted prepartum [25]. However, severe insulin resistance increases fat mobilization. Circulating NEFA are used as an indirect measure of mobilization of triacylglycerol from adipose tissue. Thus, lower responses to insulin in cows may promote greater concentrations of NEFA and BHBA, which may induce fatty liver and ketosis. Our own and other studies have demonstrated that cows overfed prepartum had higher NEFA and BHBA concentration during early lactation [10,19]. In our previous study, the HD cows lost numerically more BCS and BW postpartum [3].

Glucagon promotes the capacity of hepatic gluconeogenesis in early lactating cows [26]. In the current study, HD cows had a lower concentration of glucagon than the MD and LD cows during the first two wk of lactation, which indicated an improved gluconeogenic status and may be related to the increased production of lactose. In our previous study, the average lactose production for the LD and HD cows was 1.72 and 1.58 kg/d, respectively, during the first 3 wk of lactation.

### Expression of genes related to lipolysis and gluconeogenesis

Adipose tissue mobilization is essential for high producing dairy cows to meet the energy requirement for milk production in early lactation. HSL is the rate-limiting enzyme in lipolysis. In the present study, the mRNA abundance of HSL in adipose tissue was increased markedly for all the three groups from −7 to 14 d relative to calving, and it was significantly higher for HD cows than LD and MD cows at 7 DIM. These results indicated that, with the prolongation of early lactation, the amount of adipose tissue mobilization increased rapidly, especially for cows fed a high energy density diet prepartum. Over conditioned cows have larger adipocytes and are predisposed to excessive mobilization of body fat due to a higher basal and stimulated lipolytic activity of large adipocytes [27]. Jaakson et al [28] also found that over conditioning during the dry period was related to intensified lipid mobilization at the beginning of lactation. Our previous study demonstrated that the cows fed a high energy density diet showed numerically more BCS and BW prepartum, and lost numerically more BCS and BW postpartum [3].

Leptin is a hormone-inducing protein secreted by adipose tissue that plays an important role in EB and body fat stability [29]. In the present study, the mRNA abundance of leptin in adipose tissue was increased markedly for all cows from −7 to 14 d relative to calving, which was similar to the effect of HSL. These results also suggested that adipose tissue mobilization increases rapidly during early lactation, but the prepartum dietary energy density has no effect on mRNA abundance of leptin postpartum. Another study showed that prepartum energy level had only transient effects on the serum leptin concentration in dairy cows during the transition period [30].

CPT1 and PPARα are involved in hepatic fatty acid oxidation. Their mRNA abundance is positively correlated with circulating NEFA and BHBA concentrations [31]. In the current study, although the high energy diet increased concentrations of NEFA and BHBA postpartum, it did not cause changes in mRNA expression of CPT1 and PPARα. Other research has also shown that prepartum energy supplementation does not have an effect on liver triglyceride accumulation, nor on the mRNA abundance of CPT1 and PPARα [32]. If excessive lipolysis occurs during early lactation the oxidative capacity of the liver can be exceeded, resulting in liver triglyceride accumulation and ketosis, which reduces hepatic gluconeogenic capacity.

The PC catalyzes the conversion of pyruvate to oxaloacetate, and cytosolic PEPCK catalyzes the conversion of oxaloacetate to phosphoenolpyruvate. Both enzymes have been shown to respond to the onset of lactation. In the present study, the mRNA abundance of the two enzymes increased markedly for the cows given all three treatments at 3 DIM compared with −7 d relative to calving. The prepartum low energy diet had a tendency to increase hepatic PC mRNA expression, which was consistent with higher postpartum blood glucose concentration and lower NEFA and BHBA concentrations in the LD cows. Other research has also shown that the abundance of hepatic PC mRNA at 1 d was elevated significantly when compared with −14 d relative to calving, but there was no significant difference for that of hepatic PEPCK [33]. There was little difference in the abundance of PEPCK mRNA between −7 d and 14 d relative to calving. Laguna et al [34] reported similar findings for the mRNA abundance of cytosolic PEPCK in dairy cows during the transition period. These results indicate that PC is more susceptible to EB than PEPCK, and that PEPCK is a more important rate-limiting enzyme involved in glucose production than PC. Glucagon increases the mRNA expression of gluconeogenic enzymes in the liver of early lactating cows [26]. In the current study, HD cows had a lower concentration of blood glucagon than MD and LD cows in the first 2 wk of lactation, and this was consistent with the lower mRNA abundance of PC during early lactation.

## CONCLUSION

*Ad libitum* feeding of a reduced energy density diet during the close-up period decreased the mRNA abundance of HSL in adipose tissue, decreased the concentrations of circulating insulin, glucagon, NEFA and BHBA, and improved the mRNA abundance of hepatic PC in early lactation. These results suggested that a low energy density diet was beneficial in controlling lipolysis and promoting the capacity for hepatic gluconeogenesis postpartum. Whether the mRNA abundance of hepatic PEPCK is not susceptible to regulation by NEB needs more in-depth research.

## Figures and Tables

**Table 1:** Composition and analysis of diets fed to Holstein cows during the dry and lactating periods

Item	Far-off diet	Close-up diets			Lactation diet1)

		HD	MD	LD	
Ingredients (% DM)					
Leymus chinensis hay	42.8	13.3	21.7	31.3	-
Alfalfa hay	-	8.4	8.4	8.4	17.2
Whole corn silage	24.7	21.0	21.1	21.0	21.0
Corn	1.2	23.2	14.4	2.0	20.0
Extruded soybean	-	-	-	-	2.6
Soybean meal	2.1	4.5	4.0	3.7	5.8
Rapeseed meal	4.5	4.0	4.0	4.0	1.3
Cottonseed meal	5.0	4.0	4.3	4.5	1.3
Apple pomace	9.0	5.6	7.4	11.3	2.5
Whole cottonseed	-	3.3	3.3	3.3	10.4
Distillers dried grains with soluble	4.1	4.0	4.0	4.0	10.0
Palm kernel meal	5.1	3.3	3.3	3.3	-
Beet pulp meal	-	-	-	-	3.5
Calcium carbonate	1.2	1.9	1.9	1.9	1.0
Close-up premix2)	0.1	0.4	0.4	0.4	-
Lactation premix3)	-	-	-	-	0.5
Bergafat4)	-	2.2	0.9	-	1.6
Yeast culture5)	0.2	0.9	0.9	0.9	0.4
Sodium bicarbonate	-	-	-	-	0.9
Analysis					
DM (%)	51.9	51.5	51.5	51.6	52.1
CP (% of DM)	12.6	14.0	14.0	14.0	18.2
NDF (% of DM)	53.3	37.4	42.9	49.8	31.7
ADF (% of DM)	32.4	23.2	26.8	31.5	19.8
NFC (% of DM)	22.8	35.0	30.4	23.9	36.9
Crude fat (% of DM)	3.1	5.6	4.3	3.3	5.7
NE_L_ (MJ/kg DM)	5.4	6.8	6.2	5.4	7.2
ME (MJ/kg DM)	8.7	10.7	10.0	8.8	11.1

HD, high energy density diet; MD, medium energy density diet; LD, low energy density diet; DM, dry matter; CP, crude protein; NDF, neutral detergent fiber; ADF, acid detergent fiber; NFC, nonfiber carbohydrate; NE_L_, net energy for lactation; ME, metabolizable energy.

1) Lactation diet was fed to cows from parturition to 70 d in milk.

2) Close-up premix contained (per kg of premix; DM basis): 2,200,000 IU of vitamin A, 550,000 IU of vitamin D_3_, 20,000 IU of vitamin E, 2,000 mg of vitamin PP, 3,750 mg Cu, 5,720 mg Mn, 14,850 mg Zn, 150 mg I, 180 mg Se, 120 mg Co.

3) Lactation premix contained (per kg of premix; DM basis): 1,000,000 IU of vitamin A, 280,000 IU of vitamin D_3_, 10,000 IU of vitamin E, 1,000 mg of vitamin PP, 3,250 mg Cu, 4,800 mg Mn, 12,850 mg Zn, 140 mg I, 150 mg Se, 110 mg Co.

4) Fractionated palm fatty acids (Berg+Schmidt, Hamburg, Germany).

5) Diamond V XP, Diamond V Mills, Inc..

**Table 2:** Sequences of the primers used for quantitative polymerase chain reaction in adipose tissue and liver

Primer	Primer sequence (5′-3′)	Product size (bp)
β-actin	F: TCATCACCATCGGCAATGAG	378
	R: CATCGTACTCCTGCTTGCTGA	
RPL19	F: AGGGTACTGCCAATGCTTTAATG	113
	R: CATGTGGCGGTCAATCTTCTT	
PC	F: CCCACAGCTTCAACAAACTCTT	236
	R: GATGTCCATGCCATTCTCCTT	
PEPCK	F: CCAACTCACGGTTCTGCACT	225
	R: GCATGATGACTTTGCCCTTGT	
CPT1	F: GCAGCGTTCTTCGTGACGTTA	115
	R: ACCTGTCGAAACACCTGCCAT	
PPARα	F: GAGCTGGACGACAGTGATATTT	144
	R: ATGGTTGTTCTGTAGGTGGAGT	
HSL	F: ACGAGCCTTACCTCAAGAGCTG	124
	R: CAGCAGTAGGCATAGGAGCACTC	
PPARγ	F: CCCTTTGGTGACTTTATGGAG	155
	R: TGTATGTCCTCAATGGGCTTC	
InsR	F: AGCTGGAGGAGTCCTCGTTC	113
	R: TGTCACATTCCCCACGTCA	
LEP	F: CTTTGGCCCTATCTGTCTTACGT	76
	R: TCTTGATGAGGGTTTTGGTGTCA	

RPL19, ribosomal protein L 19; PC, pyruvate carboxykinase; PEPCK, phosphoenolpyruvate carboxykinase; CPT1, carnitine palmitoyltransferase-1; PPARα, peroxisome proliferator-activated receptor-α; HSL, hormone sensitive lipase; PPARγ, peroxisome proliferator-activated receptor-γ; InsR, insulin receptor; LEP, leptin.

**Table 3:** Effect of close-up dietary energy density on blood metabolites and hormones in Holstein cows

Item	Dietary treatments			SEM	p-value		
	
	HD	MD	LD		Diets	Time	Interaction
Glucose (mmol/L)							
−2 to −1 wk	4.18	4.15	4.00	0.05	0.22	<0.001	0.60
0 d	7.92	7.64	6.98	0.22	0.36	-	-
1 to 2 wk	3.27^b^	3.79^a^	3.65^ab^	0.08	0.02	0.98	0.59
1 to 4 wk	3.47^b^	3.71^a^	3.67^ab^	0.04	0.04	0.71	0.13
Isulin (lu/mL)							
−2 to −1 wk	16.56^a^	16.16^a^	14.43^b^	0.41	0.01	0.02	0.98
0 d	4.94	5.01	5.62	0.22	0.40	-	-
1 to 2 wk	6.51^b^	8.42^a^	8.77^a^	0.52	0.01	0.008	0.046
1 to 4 wk	10.06	10.59	10.88	0.65	0.66	<0.001	0.26
Glucagon (pg/mL)							
−2 to −1 wk	134.86	122.43	117.47	5.81	0.15	<0.001	0.14
0 d	144.06	150.91	130.29	8.59	0.46	-	-
1 to 2 wk	163.97^b^	202.48^a^	207.17^a^	10.30	0.01	0.23	0.96
1 to 4 wk	182.10	197.21	190.42	8.21	0.44	<0.001	0.04
NEFA (μEq/L)							
−2 to −1 wk	222.78^b^	251.77^ab^	283.50^a^	11.89	0.005	<0.001	0.59
0 d	463.88	497.23	541.44	25.79	0.49	-	-
1 to 2 wk	605.67^a^	526.99^ab^	464.78^b^	32.62	0.02	0.15	0.92
1 to 4 wk	496.03^a^	452.39^ab^	399.47^b^	20.15	0.01	<0.001	0.82
BHBA (mmol/L)							
−2 to −1 wk	0.57	0.59	0.51	0.02	0.15	0.02	0.48
0 d	0.58	0.55	0.65	0.3	0.72	-	-
1 to 2 wk	0.67^a^	0.52^b^	0.54^b^	0.02	0.03	0.69	0.61
1 to 4 wk	0.66	0.59	0.60	0.02	0.32	0.11	0.58
PUN (mmol/L)							
−2 to −1 wk	3.43	3.67	4.12	0.15	0.19	<0.001	0.52
0 d	3.53	3.44	3.18	0.21	0.56	-	-
1 to 2 wk	4.08	4.21	3.86	0.18	0.72	0.07	0.43
1 to 4 wk	4.54	4.47	4.24	0.14	0.65	<0.001	0.82

HD, high energy density diet; MD, medium energy density diet; LD, low energy density diet; SEM, standard error of the mean; NEFA, nonesterified fatty acids; BHBA, β-hydroxybutyrate; PUN, plasma urea nitrogen.

a,b Values within a row with different superscripts differ significantly at p<0.05.

**Table 4:** Effect of close-up dietary energy density on mRNA abundance of genes related to mobilization of adipose tissue in Holstein cows

Item	Day relative to parturition (d)		

	−7	3	14
HSL			
HD	0.26	0.95^a^	3.30
MD	0.23	0.48^b^	3.21
LD	0.31	0.51^b^	2.93
SEM	0.03	0.08	0.23
p-value	0.53	0.001	0.83
LEP			
HD	0.08	0.14	1.13
MD	0.08	0.08	0.92
LD	0.09	0.13	1.08
SEM	0.01	0.01	0.10
p-value	0.87	0.24	0.73
InsR			
HD	0.31	0.42	0.56
MD	0.40	0.46	0.53
LD	0.37	0.42	0.53
SEM	0.03	0.09	0.04
p-value	0.63	0.98	0.92
PPARγ			
HD	0.33	0.38	0.41
MD	0.31	0.34	0.34
LD	0.34	0.37	0.39
SEM	0.03	0.03	0.05
p-value	0.92	0.90	0.86

HSL, hormone sensitive lipase; HD, high energy density diet; MD, medium energy density diet; LD, low energy density diet; SEM, standard error of the mean; LEP, leptin; InsR, insulin receptor; PPARγ, peroxisome proliferator-activated receptor-γ.

a,b Values within a column with different superscripts differ significantly at p<0.05.

**Table 5:** Effect of close-up dietary energy density on mRNA abundance of genes related to hepatic gluconeogenesis in Holstein cows

Item	Day relative to parturition (d)		

	−7	3	14
PEPCK			
HD	0.28	0.32	0.29
MD	0.21	0.31	0.19
LD	0.17	0.42	0.22
SEM	0.03	0.04	0.03
p-value	0.25	0.39	0.53
CPT1			
HD	0.51	0.61	0.54
MD	0.50	0.57	0.53
LD	0.56	0.80	0.61
SEM	0.04	0.11	0.04
p-value	0.88	0.74	0.72
PC			
HD	0.29	0.52	0.68
MD	0.31	0.70	0.59
LD	0.42	0.81	0.64
SEM	0.04	0.06	0.04
p-value	0.34	0.08	0.71
PPARα			
HD	0.57	0.72	0.55
MD	0.46	0.71	0.54
LD	0.45	0.80	0.64
SEM	0.05	0.07	0.06
p-value	0.60	0.87	0.82

PEPCK, phosphoenolpyruvate carboxykinase; HD, high energy density diet; MD, medium energy density diet; LD, low energy density diet; SEM, standard error of the mean; CPT1, carnitine palmitoyltransferase-1; PC, pyruvate carboxykinase; PPARα, peroxisome proliferator-activated receptor-α.
